# One-dimensional and comprehensive two-dimensional gas chromatographic approaches for the characterization of post-consumer recycled plastic materials

**DOI:** 10.1007/s00216-023-04599-6

**Published:** 2023-02-23

**Authors:** Andrea Hochegger, Sebastiano Pantò, Nick Jones, Erich Leitner

**Affiliations:** 1 University of Technology Graz, Institute of Analytical Chemistry and Food Chemistry, Stremayrgasse 9/II, 8010 Graz, Austria; 2 LECO European Application and Technology Center (EATC), Berlin, Germany

**Keywords:** GC × GC-ToFMS, Post-consumer recycled plastics, Gas chromatography, Mass spectrometry

## Abstract

In September 2022, the European Commission published its new regulation on recycled plastic materials for food contact. It allows newly developed, non-authorized technologies and approaches, or so-called novel technologies, to be deployed in the field to generate the data needed for establishing regulatory and/or fit for purpose processes. The data shall be generated by using suitable methods, but the regulation does not give a more detailed description on those. In this study, commercially purchased buckets made of post-consumer recycled polypropylene were screened, using a number of different analytical approaches. Sample preparation methods, analysis techniques, and the data and information generated were compared. The results clearly demonstrate the need for a detailed characterization of such materials and the advantages and disadvantages of the analysis using conventional gas chromatography with flame ionization detection and mass spectrometery as well as two-dimensional comprehensive gas chromatography with time of flight mass spectrometry.

**Graphical Abstract:**

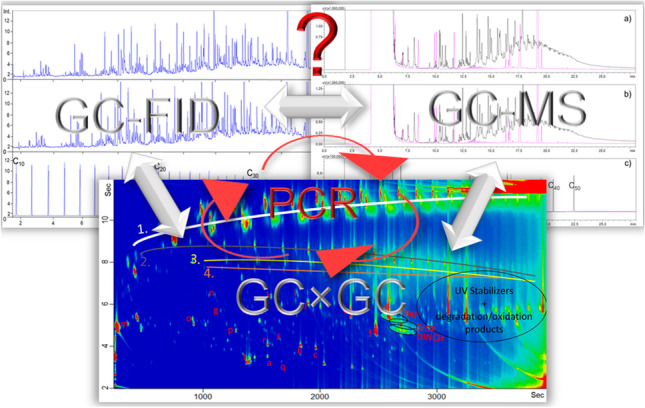

## Introduction

Europe’s vision of the new circular economy regarding packaging materials brings opportunities and challenges. The vision requires that 65% of all packaging materials shall be reusable or effectively recyclable by 2025, and that 75% will be by 2030. This means that 55% of plastic packaging and 75% of paper and cardboard packaging will need to be recycled by 2025, and by 2030, all plastic packaging produced in the EU market will need to be reusable or recycled in a cost-effective manner [1, 2]. Subsequently, the development and deployment of highly efficient sorting procedures and recycling processes are therefore needed to enable production of high-quality recycled products, as well as analytical strategies for their characterization and safety evaluation. One of the main applications for recycled plastic-based packaging materials is the food packaging sector. A key requirement is to guarantee that processes used result in products that are safe for food contact and therefore for the consumer. Thereby, the principle of Regulation (EC) No 1935/2004 that food contact materials should not change the food in any unacceptable way, neither in terms of taste or quality nor in terms of contaminations, is indisputable [3].

The current state of the art and knowledge regarding sorting, recycling technologies, challenge, and compliance testing were lately discussed in several reviews [4–8]. All of them concluded that most knowledge is available for post-consumer recycled PET, since it is one of the most often studied and also used materials. More challenging is the situation regarding polyolefins, since their properties differ significantly from PET: the sorption and migration potential of polyolefins is high compared with PET [9] leading to the need to measure a bigger range of physical properties such as molecular weights. It is well known that decontamination efficiency decreases with increasing molecular weight. Therefore, special attention must be paid to higher molecular weight substances. Furthermore, the contamination levels of polyolefins may be higher due to the better sorption properties and due to the wide range of application and use of polyolefin packaging materials [4–7].

As a consequence, to adapt to the new situation regarding the circular economy and the higher need and therefore higher diversity of processes, technologies, and products being developed lately, the European Commission published its new regulation on recycled plastic materials and articles intended to come into contact with foods in September 2022 [10]. The aim of the new regulation is to adopt to the changing market situation, giving the possibility to recyclers to develop and use new technologies more easily. In relation to that, recyclers are allowed to use newly developed, non-authorized technologies, so-called novel technologies, and bring products onto the market to generate the data needed for the approval and deployment of suitable processes. To evaluate these novel technologies, various analytical methods are needed, to analyze the produced materials, to determine the decontamination efficiency of the technology and the contamination level of the final products. A list of all substances detected having a molecular weight lower than 1000 dalton (Da) shall be provided, in which at least the 20 most prominent substances shall be identified. The methods used for sampling, sample preparation, and analysis shall be appropriate for the intended purpose, with documents being available to demonstrate suitability. No further details on the methods are given, but it is accepted that a challenge test — as set down in the criteria for PET — is not appropriate for this purpose. In a challenge test, the input material is spiked with a set of known surrogates and the decontamination efficiency of the process determined afterwards by determining the residual amounts of surrogates [11]. In the literature, different sets of surrogates for challenge testing had been proposed, depending on the kind of polymer. The surrogates are compounds identified to be usual contaminants in the input material, thereby also representing a range of molecular weights and polarities (e.g., toluene 92 g/mol, chlorobenzene 113 g/mol, methyl salicylate 152 g/mol, phenylcyclohexane 160 g/mol, benzophenone 182 g/mol, di(ethylhexyl) phthalate 222 g/mol, hexachlorocyclohexane 291 g/mol, methyl stearate 298 g/mol, isopropyl stearate 326 g/mol) [4, 12]. Nevertheless, the challenge test may be an important tool to monitor a recycling process in routine manner, once it is approved and accepted to be suitable and safe. Using the determined decontamination efficiency, information on the sorption properties of the polymer, and different safety factors, the recycling technologies can be evaluated, as lately discussed [8].

The question arises, which methods may be suitable to generate the needed data. Only some publications deal with characterization of post-consumer recycled polyolefins, many being published in the 1990s [5, 13–18]. In summary, analysis of volatile substances was done using conventional gas chromatographic methods and analysis of semi-volatiles using high-performance liquid chromatography (HPLC). Gas chromatography (GC) is often used in a screening approach, either after total extraction or after headspace – solid-phase microextraction (HS-SPME). Detectors used are mass spectrometers (GC–MS) or flame ionization detectors (GC-FID). However, data on recycled polyolefins (rPO) is limited. One of the main problems identified is the limited range of molecular weight to be detected using conventional GC methods, limiting the detection of substances having high molecular weights which may be of potential concern [4, 6]. Nevertheless, Su et al. [6] reported in their study a list of more than 50 substances identified and semi-quantified in rPO samples from Spain and China, posing a potential health concern by using direct-immersion solid-phase micro-extraction (DI-SPME) connected to GC–MS and gas chromatography-quadrupole-time of flight mass spectrometry with atmospheric pressure chemical ionization.

The aim of this study was to directly compare different analysis approaches with each other in terms of the quality of data and information generated from the analysis of post-consumer recycled materials. Most promising seemed to be the use of comprehensive two-dimensional gas chromatography (GC × GC)-based techniques, which allows detailed insights into volatile substances present in post-consumer recycled plastics. It is well known that this technique has huge advantages over conventional GC, such as an increased peak capacity, higher chromatographic resolution, higher sensitivity, and structured chromatograms (allowing an easier identification of whole substances groups). Biedermann and Grob [19] discussed the named arguments and concluded GC × GC can therefore be a key and fit for purpose technique in assessing migrants from various food contact materials (FCM) [19–22].

In the present study, two buckets made from post-consumer recycled polypropylene, which were purchased in a local discounter market, were investigated. The applied method was based on the Guidance in selecting analytical techniques for identification and quantification of non-intentionally added substances (NIAS) in food contact materials [23], and aimed to create an overview on the substances present that might be of interest for human health. The information generated following either exhaustive extraction or HS-SPME as sample preparation combined with different analytical techniques was compared: conventional GC with FID as a universal detector was used for an easy quantification of total extractables, GC with electron ionization MS for identification of substances present by using conventional mass spectral databases. Online-coupled HPLC-GC-FID was used to determine the amount of saturated and aromatic hydrocarbons in the samples and GC × GC with time-of-flight mass spectrometry (ToFMS) for a detailed compositional characterization. The latter two allowed analysis of substances up to *n*-Pentacontane (*n-*C_50_). The results of the different approaches are compared with each other, taking the current recommendations and regulations into consideration.

## Materials and methods

Two different post-consumer recycled polypropylene (PP) buckets were purchased at a local discounter market in Graz, Styria, Austria, and labelled “A” and “B.” Bucket A was of gray color having about 5 L of volume, Bucket B being of black color, having about 12 L volume. No recommendations or information about restrictions in usage was given.

For sample preparation, two approaches were used: a quick and easy exhaustive extraction was performed to determine the amount of volatile extractables using GC-FID. Additionally, to get a detailed overview on the types of chemical compounds and their tentative identification, analysis of the extracts was performed using GC × GC-TOF–MS. The buckets were first cut into small pieces using pincers, and then grounded to fine powder in a cryogenic mill. Total extraction of 5 g of the ground materials was done in glass vials, using 10 mL of Cyclohexane (ROTRISOLV® Pestilyse® ≥ 99.5%, Carl Roth GmbH + Co. KG, Karlsruhe, Germany) in an ultrasonic bath at 60 °C for 1 h. Afterwards, the sample material was allowed to settle over night at room temperature.

To determine the sum of volatile compounds, an aliquot of this extract was measured after addition of 10 µL of an internal standard mix for quantification directly on a GC-FID system. The internal standard mix consisted of deuterated n-Undecane-d_22_ (d-C_10_; 99atom% deuterium, Sigma-Aldrich, Merck KGaA, Darmstadt, Germany), n-Dodecane-d_26_ (d-C_12_; EURISOTOP SAS, Saint-Aubin, France), n-Hexadecane-d_16_ (d-C_16_; 98% purchased at Cambridge Isotope Laboratories, Inc. (Tewksbury, MA, USA)), and Toluol-d_8_ at a concentration of 25 mg/L each in Methanol (for HPLC–MS, Fisher Chemical, Fisher Scientific GmbH, Schwerte, Germany). The analysis was performed using a Hewlett Packard 6890 Series GC system equipped with an Optima delta-6 capillary column (7.5 m × 0.1 mm × 0.10 µm, Macherey–Nagel, Germany). The oven was programmed to 45 °C (hold 1 min) and raised at 15 °C/min to 300 °C (hold 3 min). Carrier gas was hydrogen with a constant flow of 48 cm/s. Aliquots of 1 μL were injected with a split ratio of 1:20; the injection port temperature was set to 280 °C. The detector was heated to 320 °C, air flow was 450 mL/min, hydrogen flow was 40 mL/min, and make up gas was nitrogen with a flow of 25 mL/min. Data evaluation was done using “GC ChemStation” version B.04.03 [16]. Data evaluation was done by comparing the areas of the added internal standards with the area of all detected natively present substances in the samples. Differentiation of deuterated and native substances was possible via a slight retention time shift, identified by measuring dilutions of single compounds.

To generate a more detailed overview of the substances encountered, the total extract was diluted 1:10 using cyclohexane and aliquots of 1µL injected without any further sample preparation into the GC × GC-ToFMS system. The system was a PEGASUS® BT 4D GC × GC-TOFMS (LECO, St. Joseph, MI, USA), controlled by the Leco “ChromaTOF” software in version 5.51.50. The instrument consisted of a 7890B gas chromatograph (Agilent Technologies, Waldbronn, Germany) equipped with a cool on-column injector, an Agilent 7693A autosampler, a secondary internal oven, a quad-jet dual stage thermal modulator, and a time-of-flight mass spectrometer. On-column injection was performed onto a 0.3 m × 0.53 mm i.d. guard column, connected via an Ultimate Union (Agilent, Technologies, Waldbronn, Germany) to a 15 m × 0.25 mm i.d. × 0.15 μm Rxi-17Sil MS (Restek Corporation, Bellefonte, USA) first dimension column, which was further connected via a SilTite® µ-Union (Trajan Scientific and Medical, Victoria, Australia) to a 1.6 m × 0.18 mm i.d. × 0.18 μm ZB-1HT Inferno® (Phenomenex, CA, USA) second dimension column. The columns were temperature programmed from 40 °C (hold 1 min) to 360 °C at 5 °C/min (hold 1 min) with a secondary oven offset of + 15 °C. The modulator offset was also set to + 15 °C. Helium was used as a carrier gas with a 1^st^ dimension linear velocity of 39 cm/s and a 2^nd^ dimension linear velocity of 125 cm/s. Modulation time was 10 s, with 3 s hot jet and 2 s cold jet time. Spectra were collected in the m/z range from 50 to 700, with a data acquisition rate of 100 spectra/s. The ion source was set to 250 °C, the transfer line to 340 °C. The detector voltage was relative to tune (2.1 kV) and was applied after the solvent delay of 240 s.

To identify the most prominent substances in an easier, faster, and more conventional approach, head-space solid-phase microextraction (HS-SPME) with GC–MS detection of the cut buckets (without grounding) was performed. 250 mg of the cut samples was weighed into a 20-mL glass vial, a glass magnetic stir bar and 10µL aliquots of the internal standard mix for semi-quantification were also added as described above for GC-FID analysis. The vials were closed and extractions were performed at 80 °C for 20 min using a 2 cm DVB/Carboxen/PDMS fiber. Desorptions were performed in the injector port at 270 °C. Gas chromatographic separations were performed using a Shimadzu GC2010 system equipped with a Rxi-5Sil MS capillary column (30 m × 0.25 mm × 0.1 µm). Helium was used as carrier gas in linear velocity flow control mode, with a flow of 35 cm/s. The split ratio was 1:5; the septum purge flow was 6 mL/min. Initial oven temperature was − 10 °C (hold 1 min). Oven cooling was attained by the use of liquid nitrogen. The temperature was raised at 8 °C/min to 270 °C (hold 1 min). Detection was performed using a GCMS-QP2020 PLUS mass selective detector. MS fragmentation was generated with electron ionization (70 eV), the detector voltage was relative to tune (0.91 kV), and a mass scan range of m/z 35 to 350 was chosen, with a scan rate of 3 scans per second. The ion source was heated to 200 °C, the interface to 280 °C. The software used was “GC–MS Solution” version 4.42.

The amount of saturated and aromatic hydrocarbons being present in the samples was determined by using the online-coupled HPLC-GC-FID, after extracting 100 mg of grounded sample in *n*-Hexane (Picograde® for Residue Analysis; LGC Promochem GmbH; Wesel, Germany) for 2 h at room temperature. The HPLC was a Shimadzu LC 20AD, equipped with an Allure Silica 5 µm column (250 × 2.1 mm) as stationary phase. Gradient elution was performed by adjusting the mobile phase from a starting composition of 100% n-Hexane (flow 0.3 mL/min) to 65% n-Hexane and 35% dichloromethane (HPLC grade, ≥ 99.8% CH2Cl2, CHEM-LAB, Zedelgem, Belgium) over 2 min (hold for 4.20 min). The column was backflushed at 6.30 min with 100% dichloromethane (flow 0.5 mL/min; hold for 9 min) and reconditioned to 100% n-hexane (flow 0.5 mL/min; hold for 10 min). Flow was decreased afterwards to 0.3 mL/min until the next injection. The UV detector was equipped with a D2-lamp set at 230 nm and 40 °C cell temperature. The GC was a Shimadzu GC 2030 dual-column and dual-FID system, equipped with two guard columns (Restek MXT® Siltek (10 m × 0.53 mm id)) and two analysis columns (Restek MTX®-1 (15 m × 0.25 mm id × 0.1 µm df)). Carrier gas was hydrogen with a constant analysis pressure of 150 kPa and a start linear velocity at 60 °C of 182 cm/s. The oven was programmed to 60 °C (hold 6 min), raised at 20 °C/min to 100 °C (0 min) and followed by 35 °C/min to 370 °C (9.29 min). The LC-GC interface was controlled by “Chronect-LC-GC” by Axel-Semrau. Data evaluation was done using “LabSolutions” of Shimadzu Corporation for LC and GC data in version 5.92.

## Results and discussion

The new regulation on recycled plastic materials for food contact requires the use of analytical “methods” for the characterization of input materials and final products. In this study, different approaches for the characterization were compared with each other. The main aim was to generate as much information as possible on the conventionally purchased buckets, based on the guidance and criteria for the detection of NIAS in FCM [23]. Samples were cryogenically milled to increase the surface area and therefore the extraction efficiency. Exhaustive extraction in cyclohexane was performed to extract and determine all potentially migrating substances with a molecular weight below 1000 Da, thereby identifying the possible migrants. Due to the high sorption of substances into polyolefins, the molecular range of analysis is highly relevant. Using gas chromatography-based techniques, the limiting factor is mainly the operational temperature limit of the capillary column used. In this study, we used a medium polar Optima-Delta 6 with a max. temperature of 360 °C for the GC-FID analysis to determine the overall amount of extracted substances. It allowed analysis of substances of different polarities, without significant discrimination up to *n*-C_38_ (molecular weight 535 Da). In comparison, the online coupled HPLC-GC-FID is with its used steel columns optimized for the analysis of high boiling compounds reaching at least *n*-C_50_ (703 Da). The 2D approach using GC × GC-ToF–MS was adapted to the range of the HPLC-GC-FID system. A cool-on column injector is used, preventing discrimination of high boiling substances in the injector, and the column selection was made due to temperature stability (the ZB-1HT Inferno® in the second dimension has a maximum temperature limit of 430 °C), which is essential due to the use of the high temperature offsets in the modulator. The combination with the 17Sil-MS column is optimized for the separation of aliphatic and aromatic substances with a focus on optimized separation and peak shape for the aromatic fraction, which is highly important for these complex samples.

A completely different approach was chosen for the GC–MS analysis. HS-SPME was used instead of liquid extraction, providing a very fast and easy analysis of the most important compounds. Furthermore, the combination of HS-SPME, the Rxi-5Sil MS column used, and a temperature program starting at − 10 °C is optimized for detecting very volatile (aroma active) compounds that might be relevant for PCR materials in food contact. The results shall be discussed for each approach in more detail.

**Fig. 1:**
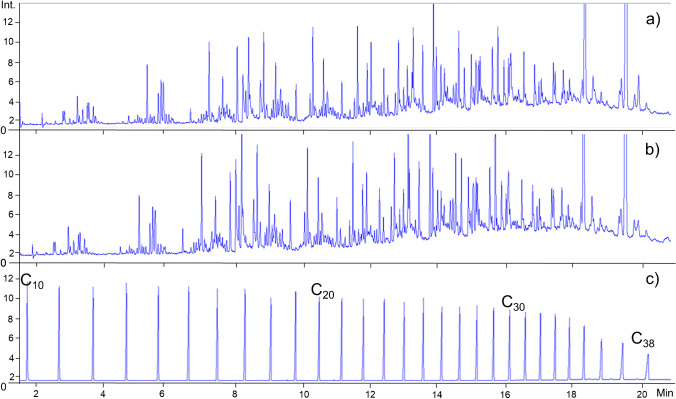
GC-FID chromatograms of purchased buckets: **a**) Sample A, 5L gray bucket; **b**) Sample B, 12L black bucket; **c**) n-Alkanes C_10_-C_38_ for determination of retention times

The analysis of the total sum of volatile compounds (retention times between *n*-Decane (C_10_) and *n-*Octatriacontane (C_38_) using GC-FID and semi-quantification via the added internal standards revealed a sum of 4.4 g and 3.8 g of compounds per kg for Buckets A and B after total extraction for 1 h at 60 °C in an ultrasonic bath, respectively (Fig. 1).

**Fig. 2:**
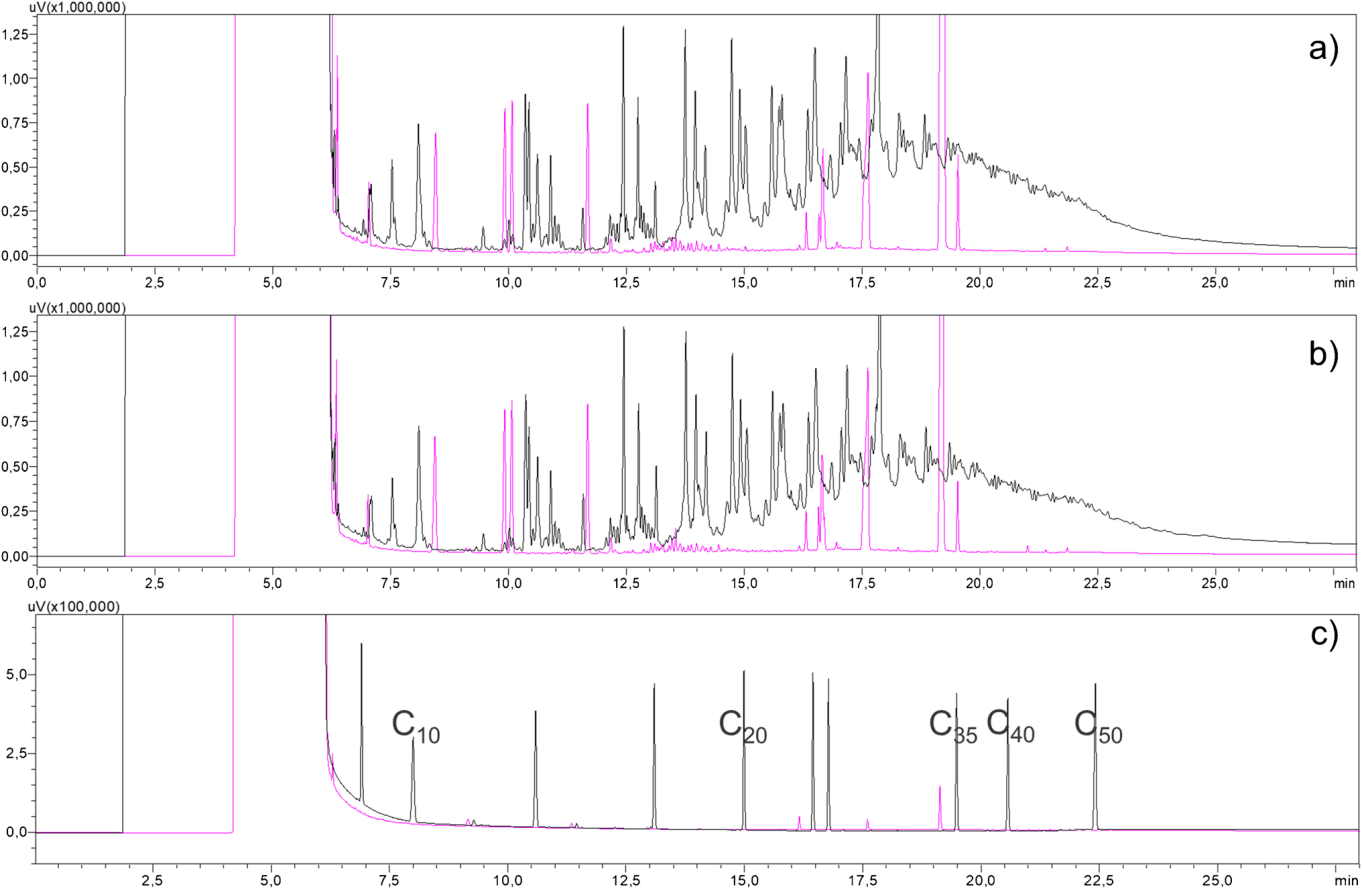
Online-coupled HPLC-GC-FID chromatograms of purchased buckets: **a**) Sample A, 5L gray bucket; **b**) Sample B, 12L black bucket; **c**) n-Alkanes for comparison of retention times

Those amounts are comparable to, e.g., the amount of substances extracted from a recycled paper or board, and may pose similar health risks [24, 25]. In terms of recycled paper and board, mineral oil hydrocarbons (MOH) are one main source of contamination. Those are routinely determined as mineral oil saturated and aromatic hydrocarbons (MOSH and MOAH) after extraction with *n*-Hexane at room temperature using the online-coupled HPLC-GC-FID [26, 27]. Since for polypropylene saturated hydrocarbons are intrinsically present, we determined the amount of saturated and aromatic hydrocarbons being present in the sample regardless of their origin. Sample A showed a content of 3.4 g/kg saturated hydrocarbons and 0.17 g/kg aromatic hydrocarbons, while Sample B had 3.1 g/kg saturated and 0.15 g/kg aromatic hydrocarbons. Both chromatograms (Fig. 2) show a typical pattern of polyolefin oligomeric saturated hydrocarbons (POSH) from the polypropylene, in the saturated hydrocarbons fraction [27].

However, the contamination found for aromatic hydrocarbons cannot be explained by their intrinsic presence. They must originate from the recycling input materials, where they could be present because of the intended use of PP as, e.g., a fuel canister, or because of unintended use (e.g., refilling of containers by consumers) or as another form of contamination due to their ubiquitous presence. The typical MOAH fraction is considered to be potentially mutagenic and carcinogenic, because of the possible presence of 3–7 ring polycyclic aromatic hydrocarbons (PAHs) [28–30]. If MOAH is detected in a food sample, more comprehensive analytical techniques shall be used for a detailed identification of substances present. As had been discussed previously, GC-FID hardly allows any identification of single substances; therefore, mass spectrometry is needed as a next step [19, 23, 31, 32].

Two different mass spectrometric-based techniques had been used: on the one hand, a conventional approach with HS-SPME coupled to GC–MS was used. This approach initially aimed to identify very volatile, aroma active compounds, which was hardly feasible due to the formation of non-resolved clusters, resulting from the many substances present. Still, it was capable to identify and semi-quantify the most prominent twenty substances, as indicated by Regulation 2022/1616 [10]. On the other hand, applying GC × GC-ToF–MS analysis to the diluted exhaustive extracts of the samples allowed significantly more information to be generated, and a more comprehensive overview of the substances and relative chemical classes present to be obtained.

HS–SPME–GC–MS resulted, as already mentioned, in many overlapping peaks, which made identification of single substances difficult. The most prominent peaks were tentatively identified by using conventional mass spectral databases and are given in Table 1. The identified substances are similar for both buckets and were assigned to following substance classes: n/iso-alkanes, degradation products of photo/UV stabilizers or plasticisers, fatty acid derivatives, and food and/or fragrance compounds. Many of those have been reported previously, including from samples that were not post-consumer recyclates. Among the most prominent species are the isomers of diacetyl benzene, which seem to be degradation products of bis(tert-butylperoxyisopropyl)benzol (BIBP), a cross-linking agent for rubbers and polyolefins [33] and dimethyl adipate, which might also be used as a plasticizer [34], 2,4-Di-*tert*-butylphenol is a known degradation product of antioxidants [6], as might be the isomers of Ethanone, 1-[4-(1-hydroxy-1-methylethyl)phenyl]- and 4-Isopropylacetophenone, but no literature references were found on these substances. 2-Ethyl-1-hexanol might be used as a monomer and starting substance, but has a specific migration limit (SML) of 0.05 mg/kg of food, according to [35]. It is present in both buckets in much higher levels. Diethylene glycol has a group restriction specification (SML(T)) of 30 mg/kg of food. Benzoic acid methyl ester might be used as a product additive without a SML [35].

**Table 1:** List of most prominent 20 substances tentatively identified in HS–SPME–GC–MS. Semi quantification of substances given in mg/kg bucket

	*Bucket A*		*Bucket B*	
	Compound	mg**/**kg	Compound	mg**/**kg
*1*	Hexanedioic acid, dimethyl ester	36.4	Hexanedioic acid, dimethyl ester	56.2
*2*	Isomer of Diacetylbenzene	22.6	Isomer of Diacetylbenzene	28.7
*3*	Isomer of Diacetylbenzene	19.1	Isomer of Ethanone, 1-[4-(1-hydroxy-1-methylethyl)phenyl]-	24.5
*4*	2,4-Di-tert-butylphenol	19.1	Isomer of Diacetylbenzene	22.1
*5*	Isomer of Ethanone, 1-[4-(1-hydroxy-1-methylethyl)phenyl]-	16.3	1,4-Benzenedicarboxylic acid, dimethyl ester	20.8
*6*	Dodecyl nonyl ether	14.4	Dodecyl nonyl ether	15.8
*7*	Isomer of Ethanone, 1-[4-(1-hydroxy-1-methylethyl)phenyl]-	14.0	Isomer of Ethanone, 1-[4-(1-hydroxy-1-methylethyl)phenyl]-	15.5
*8*	Heptadecane, 8-methyl-	11.8	Heptadecane, 8-methyl-	13.0
*9*	Benzyl Benzoate	11.7	1-Heptanol, 2,4-diethyl-	11.3
*10*	2-Ethylhexanol	11.0	Decanoic acid, methyl ester	9.97
*11*	2-Isopropyl-5-methyl-1-heptanol	10.6	Diethylene glycol	9.85
*12*	Decanoic acid, methyl ester	10.4	2-Ethylhexanol	9.53
*13*	Acetophenone	9.18	Benzyl Benzoate	9.30
*14*	Tetradecane	7.57	Benzoic acid, methyl ester	8.10
*15*	2,4-Dimethyl-1-heptene	7.52	11-Methyldodecanol	7.73
*16*	D-Limonene	7.46	Pentadecane	7.71
*17*	Pentadecane	7.38	4-Isopropylacetophenone	7.04
*18*	11-Methyldodecanol	7.31	1,4-Butanediol	6.95
*19*	Nonane, 5-(2-methylpropyl)-	7.07	4-Piperidinol, 2,2,6,6-tetramethyl-	6.39
*20*	Octanoic acid, methyl ester	6.35	Acetophenone	5.95

**Fig. 3:**
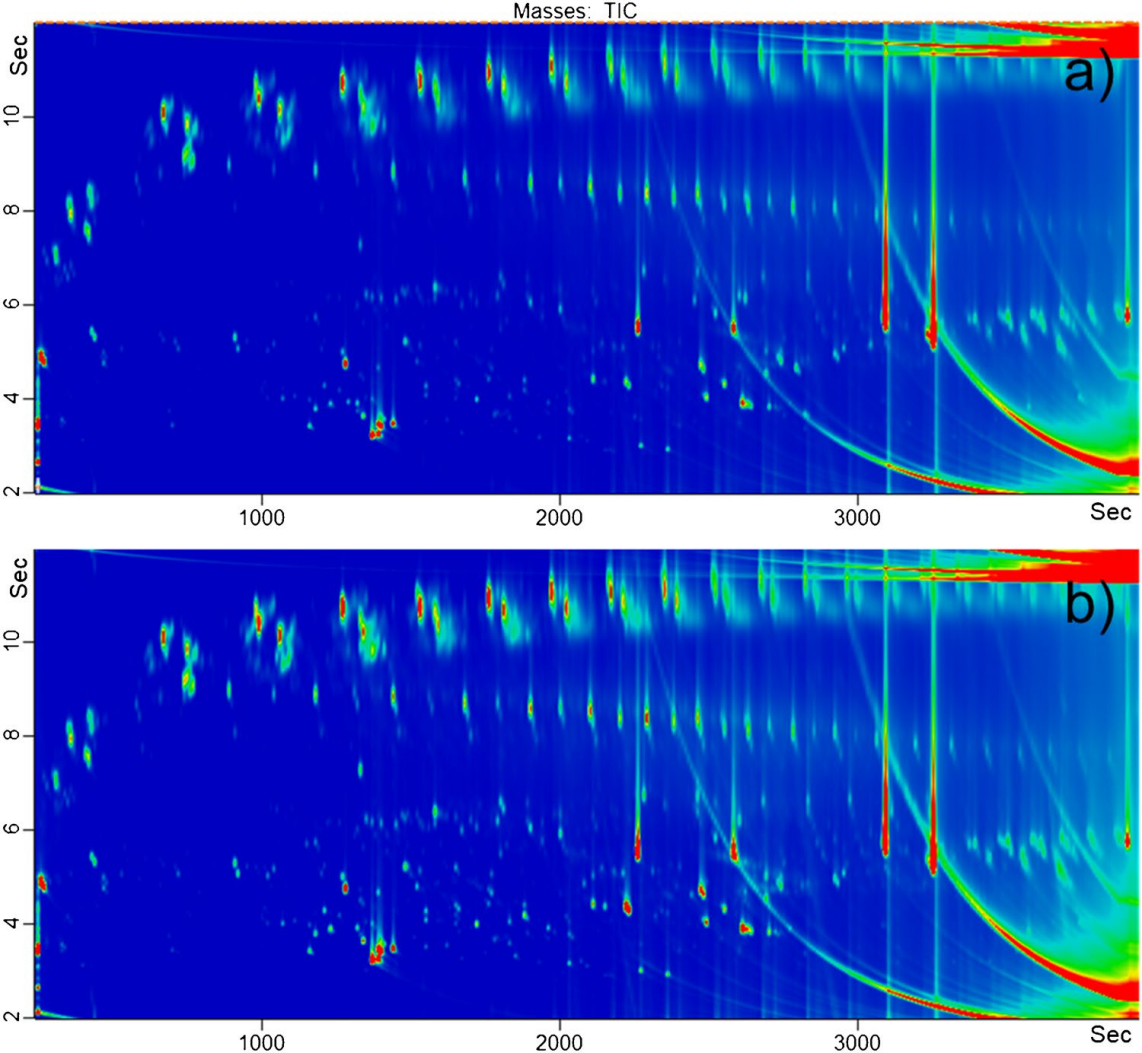
Total ion current of 2D GC × GC-ToF of purchased buckets: **a**) Sample A, 5L gray bucket; **b**) Sample B, 12L black bucket

In comparison, the information generated using the GC × GC-ToF–MS system is far richer. As discussed by Biedermann and Grob [19], the increased peak capacity and resolution in combination with the structured 2D chromatograms result into an overall better separation of the single substances and therefore easier identification. Furthermore, the huge amount of substances separated thanks to the second-dimension column demonstrates the information lost in the 1D GC analysis (Fig. 3) [19].

**Fig. 4:**
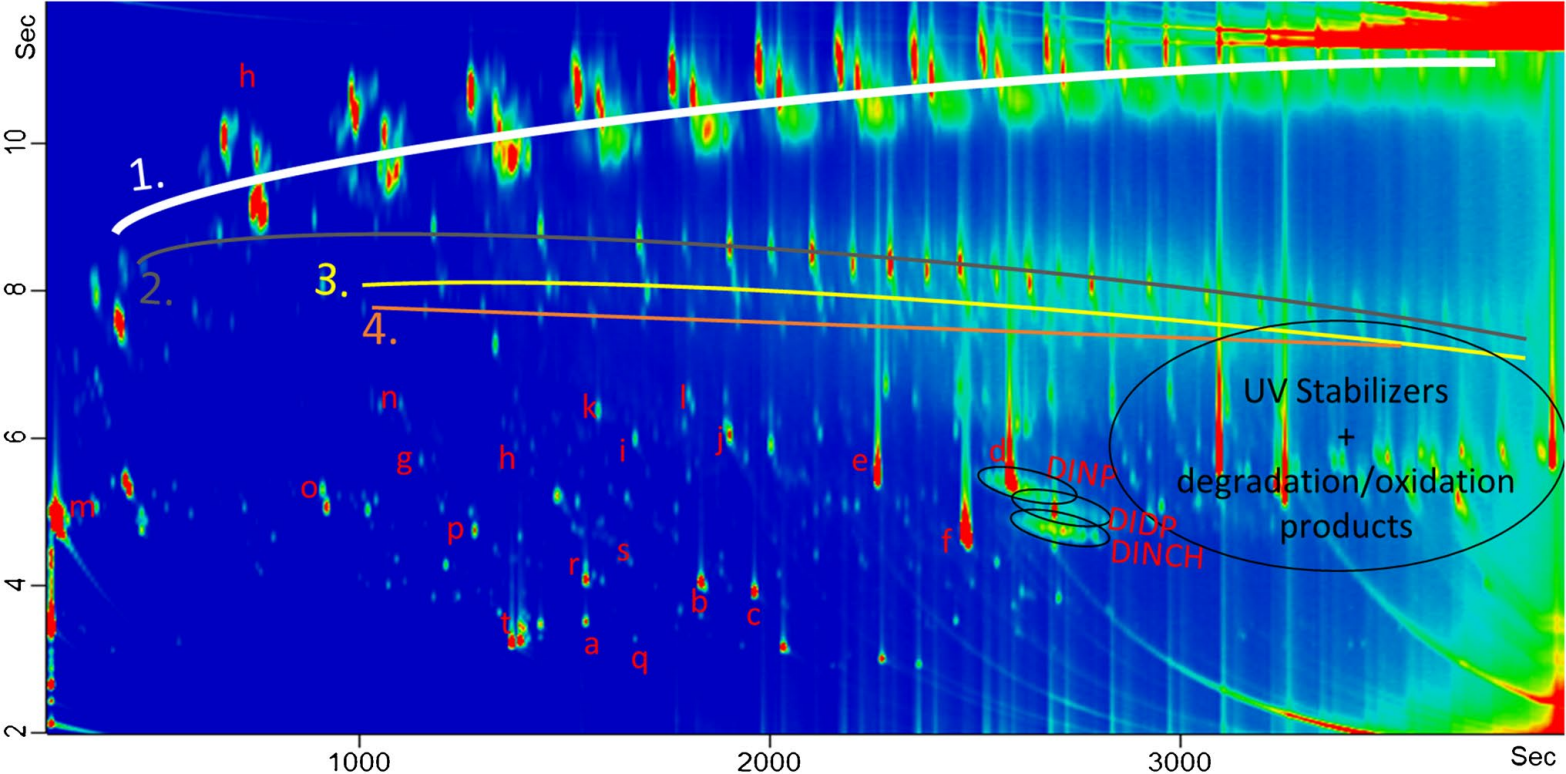
Example of identified substance groups being POSH from PP (1), n-Alkanes (2), olefins (3), n-alkyl cycloalkanes (4), DINP, DIDP, DINCH, UV stabilizers, and degradation/oxidation products, as well as identified single substances diethyl phthalate (**a**), diisobutyl phthalate (**b**), dibutyl phthalate (**c**), diisobutyl phthalate, bis(2-ethylhexyl) phthalate (**d**), bis(2-ethylhexyl) adipate (**e**), mono(2-ethylhexyl) phthalate (**f**), dodecanol (**g**), tetradecanol (**h**), hexadecanol (**i**), octadecanol (**j**), isopropyl myristate (**k**), isopropyl palmitate (**l**), limonene, linalool, or eucalyptol (in the volatile region marked with (**m**)), caryophyllenes (**n**), 2-tert-Butyl cyclohexyl acetate (**o**), 2,4-Di-tert-butylphenol (**p**), Benzophenone (**q**), 3,5-di-tert-Butyl-4-hydroxybenzaldehyde (**r**), 3,5-di-tert-Butyl-4-hydroxyacetophenone (**s**), and the isomers of Ethanone, 1,1′-(1,4-phenylene)bis- (**t**)

Again, the two buckets (A and B) look quite similar. According to proposed principles [19, 36, 37], the chromatograms were analyzed and groups of substances classified using specific mass to charge ratios to create filters. The identified classes seem to be a combination of to those being usually present in polypropylene and recycled materials, as, e.g., described for paper and board. Figure 4 gives the chromatogram of bucket A with highlighted substances and substance groups: Similar to those groups classified and discussed in [19], in the non-polar range, the saturated and olefinic clusters of POSH from PP (line 1) were clearly visible, followed by the series of n-alkanes (line 2), n-olefins (line 3), and n-alkyl cycloalkanes (line 4). Phytane and pristane, which are markers for mineral oil residues, are also present.

Among the most polar compounds in the bottom of the plot, PAH compounds such as acenaphthene, naphthalene, phenanthrene, anthracene, fluoranthene, and pyrene, as well as their slightly alkylated derivatives, were detected. Those findings are in agreement with the aromatic hydrocarbons detected using the HPLC-GC-FID analysis. The naphthalenes include the isomers of diisopropylnaphthalene, which are markers for recycled paper and board. Similar findings were also reported in virgin PP food contact materials earlier and were related to migration from printed papers giving instructions for use and being in direct contact with the PP [38].

Furthermore, clusters of plasticizers could be identified being 1,2-cyclohexanedicarboxylic acid diisononyl esters (DINCH), diisodecyl phthalates (DIDP), and diisononyl phthalate (DINP). Additionally, UV stabilizers and their degradation and oxidation products are present in the very end of the chromatogram (high boiling substances) and were identified as described earlier for virgin PP [19, 39].

Besides those structured substance classes, many other compounds are present, irregularly distributed through the GC × GC contour plot. Similar to the one-dimensional approach (Table 1), and as is mandatory for the new regulation 2022/1616, one could identify the twenty most prominent substances (besides the already characterized classes). All of those identified substances had already been reported earlier, either, e.g., as antioxidants, plasticizers, or cross-linking agents used during the production of PP or as their degradation products [19, 38, 39]. Among the 20 most prominent substances in the PP buckets are several phthalates such as diethyl phthalate (a), diisobutyl (b), dibutyl (c), bis(2-ethylhexyl) phthalate (d), and bis(2-ethylhexyl) adipate (e), which have SMLs, but also mono(2-ethylhexyl) phthalate (f), which is considered a degradation product of bis(2-ethylhexyl) phthalate. In the middle of the chromatogram oxidized hydrocarbons are present, such as alcohols (dodecanol (g), tetradecanol (h), hexadecanol (i), octadecanol (j)), isopropyl myristate (k), or isopropyl palmitate (l). Clearly visible are also different aroma active terpenes — monoterpenes such as limonene, linalool, or eucalyptol (in the volatile region marked with (m)), sesquiterpenes such as caryophyllenes (n), but also 2-tert-Butyl cyclohexyl acetate (o), which is a fragrance ingredient used in a variety of consumer products. Substances that had also been reported in earlier studies in virgin polyolefins were 2,4-Di-tert-butylphenol (p), Benzophenone (q), 3,5-di-tert-Butyl-4-hydroxybenzaldehyde (r), 3,5-di-tert-Butyl-4-hydroxyacetophenone (s), and the isomers of Ethanone, 1,1′-(1,4-phenylene)bis- (t). Because the buckets were commercially purchased without any further information on production, origin of input materials, or the recycling processes used, it is very difficult to perform a detailed analysis of all present substances and their possible origins. To identify and name every single compound being potentially of interest is just not feasible, since about 10,000 substances having a signal to noise ration > 10 were detected (excluding, e.g., column bleed), as was also discussed earlier for recycled paper and board [21].

In conclusion, thousands of substances were detected using GC × GC-ToF–MS, belonging to aliphatic saturated and unsaturated hydrocarbons, alcohols, acids and esters with aliphatic chains (including phthalates), aromatic hydrocarbons, fragrance and flavor compounds (terpenes), antioxidants, degradation fragments, and many more. Most of those substances and substance groups had been found earlier also in virgin material using GC × GC-based techniques, but to our knowledge, such a complex mixture has never been completely characterized [4, 38, 39].

In conclusion, there is a strong need for profound characterization of the whole recycling procedure — from the input materials, to the decontamination efficiency to the final product — as now required by the new Regulation 2022/1616. However, the choice of which methods are best to generate this data is still to be defined. This study clearly presents the advantages and disadvantages of the analytical techniques evaluated. GC-FID enables a quick and easy determination of the overall amount of volatile substances present in a sample, after exhaustive extraction, while the online-coupled HPLC-GC-FID allows for a further separation and quantification of the saturated and aromatic hydrocarbons. However, the FID does not allow detailed or confident identification of substances present. Therefore, mass spectrometry techniques are needed. Again, two approaches were used: HS–SPME–GC–MS is fast and allows the analysis of volatile substances without extensive sample manipulation. However, HS-SPME is strongly limited by volatility and has limited sample capacity. Also, due to the complex sample matrix, unresolved, complex humps are observed. In comparison, using GC × GC-ToF–MS after exhaustive extraction is much more powerful. The system setup used allowed the analysis of substances in the range of C_10_–C_50_, and provided much greater separation and detection of substances present, and thus much richer information, as discussed above. Easy data evaluation can be performed by applying classification techniques, thereby providing a powerful technique for routine analysis of complex matrices.

The extraction and instrumental analysis approaches applied clearly demonstrate the need for detailed and comprehensive methods of analysis for post-consumer recycled plastics. Among the detected peaks, many are regulated or of concern (e.g., have a specific migration limit or are classified as carcinogenic, mutagenic, and reprotoxic (CRM) substance). Therefore, further and more detailed studies are needed, including migration experiments to evaluate the safety of such materials and related products. In terms of methods to be used for the evaluation of recycling technologies, it is questionable, if a simple 1D GC–MS run, identifying the most prominent 20 peaks, is suitable to generate the required data. In the case of the two buckets, miniaturized Ames tests were performed, showing that both buckets are DNA-reactive (personal communication, data not shown), which was not apparent from the 1D GC analysis performed. In comparison, GC × GC-TOF–MS analysis showed the presence of many substances of possible concern. However, a detailed evaluation of those results needs to be performed, also considering other PCR samples in further studies, to determine if such contaminations and results are common for those kinds of material and if there are possibilities to remove them.
